# Profiles of direct oral anticoagulants and clinical usage—dosage and dose regimen differences

**DOI:** 10.1186/s40560-016-0144-5

**Published:** 2016-03-10

**Authors:** Masahiro Ieko, Sumiyoshi Naitoh, Mika Yoshida, Nobuhiko Takahashi

**Affiliations:** Department of Internal Medicine, School of Dentistry, Health Sciences University of Hokkaido, 1757-Kanazawa, Ishikari-Tobetsu, Hokkaido 061-0293 Japan; Division of Clinical Laboratory, Health Sciences University of Hokkaido Hospital, 2-5, Ainosato, Kita-ku, Sapporo, Hokkaido 002-8072 Japan

**Keywords:** Anticoagulant therapy, Direct oral anticoagulant (DOAC), Warfarin, Stroke prevention in atrial fibrillation (SPAF), Relative potency

## Abstract

The availability of direct oral anticoagulants (DOACs) has caused a paradigm shift in thrombosis management. DOAC profiles do not differ greatly, though they are quite different from that of warfarin, whereas their dosage and dose regimens are not consistent. The direct thrombin inhibitor dabigatran seems to obstruct tenase by inhibiting thrombin generated in the initial phase and feedback to the amplification phase of cell-based coagulation reactions. Factor Xa inhibitors (rivaroxaban, apixaban, edoxaban) mainly inhibit factor Xa activity of the prothrombinase complex in the propagation phase. The dose regimens of these inhibitors can be classified into once (rivaroxaban, edoxaban) and twice (dabigatran, apixaban) daily. On the other hand, their plasma elimination half-life times are similar, which can be explained by differences in the type of aimed anticoagulation, such as persistent (e.g., warfarin) and intermittent (e.g., low-molecular-weight heparin). Because of the differences among DOACs, an indicator is necessary to compare them. We investigated relative potency to compare dosage and intensity by calculation of conversion using a profile comprised of molecular weight, bioavailability, protein-binding rate, inhibitory constant, and dosage. We found that the relative potencies were different, with that of apixaban higher than edoxaban (60 mg) and nearly twice that of rivaroxaban. However, dabigatran could not be evaluated with this profile, likely due to its different mode of action. These results suggest that rivaroxaban and apixaban differ in regard to anticoagulation type, as the former shows persistent and the latter intermittent anticoagulation.

## Introduction

It is not unusual for doctors in the intensive care unit (ICU) to treat patients who are taking direct oral anticoagulant (DOAC) medication. DOACs are mainly used for the prevention of cardiac embolism caused by atrial fibrillation (AF), in addition to treatment of deep vein thrombosis and pulmonary embolism. AF patients tend to be elderly and their number is increasing; thus, it is thought that the numbers of patients receiving DOACs will increase considerably in the near future. Since DOAC administration may have complicated effects on a healthy condition as well as examination results, additional information about these drugs and their use for anticoagulant therapy is considered to be necessary for physicians working in the ICU.

With the recent availability of DOAC agents (profiles summarized in Table 1), thrombosis management has entered a new era [1, 2], while warfarin has played the leading role in this field for more than five decades. Among available DOACs, dabigatran etexilate (hereafter referred to as dabigatran) is the only oral anticoagulant that functions as a thrombin inhibitor and pro-drug, while the others (rivaroxaban, apixaban, edoxaban) function as activated factor X (FXa) inhibitors in an active form. These drugs vary in regard to bioavailability, renal excretion rate, and liver metabolism, while their plasma elimination half-life is comparable at around 12 h. Furthermore, their short acting profiles and modes of action are quite different from those of warfarin.

**Table 1:** Characteristics of direct oral anticoagulants

	Dabigatran	Rivaroxaban	Edoxaban	Apixaban
Inhibition target	Factor IIa	Factor Xa	Factor Xa	Factor Xa
Pro-drug	Yes	No	No	No
Liver metabolism	ca. 20 %	ca. 66 %	NR	NR
Renal excretion rate	80 %	36 %	35 %	25 %
Elimination half-life	14–17 h	7–11 h	9–11 h	10–14 h
Bioavailability	6.5 %	80–100 %	50 %	50 %
Interaction	P-gp	3A4/2 J2, P-gp	3A4, P-gp	3A4, P-gp
Dosage form	Capsule	Tablet	Tablet	Tablet

*IIa* thrombin, *Ca.* approximately, *NR* not reported, *P-gp* p-glycoprotein (inhibitor), *3A4/2J2* cytochrome P450 3A4/2J2 (inhibitor), *3A4* cytochrome P450 3A4 (inhibitor)

Dabigatran and rivaroxaban were approved for clinical use as DOACs in European countries in 2008, after which edoxaban was approved in Japan and apixaban in Europe in 2011. These four agents are now available for clinical use to prevent venous thromboembolism (VTE) and mainly administered after major orthopedic surgery. In addition, they have received approval for cases of stroke prevention in atrial fibrillation (SPAF), while edoxaban, rivaroxaban, and apixaban have been approved for the treatment of acute deep vein thrombosis (DVT) and pulmonary thromboembolism (PTE). For SPAF cases, rivaroxaban and edoxaban, which have a relatively shorter half-life among DOACs, are administered once daily, while dabigatran and apixaban, which have a relatively longer half-life, are given in twice-daily regimens. When considering their half-life values, these administration protocols do not appear to be ideal. Although the efficacy and safety of all available DOACs have been demonstrated in clinical studies, the rationale for their dose regimens is unclear, though an indicator useful for comparing them has not been previously reported. In the present study, we attempted to develop such an indicator using a profile comprised of molecular weight, bioavailability, protein-binding rate, inhibitory constant, and dosage and then investigated the relative potency of each DOAC to compare dosage and intensity.

### Inhibitory effects of DOACs in cell-based coagulation reactions

**Fig. 1:**
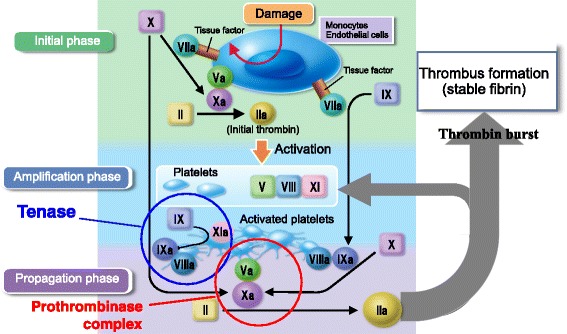
Cell-based coagulation reaction. Tissue factor is revealed in endothelial cells or peripheral monocytes by physicochemical coagulation stimulation, resulting in the formation of a small amount of thrombin, initial thrombin (initial phase). Initial thrombin activates nearby platelets and coagulation factors. Tenase (X-ase) is then formed on the negative-charged phospholipid membrane of activated platelets, and FXa is converted from FX. FXa forms a prothrombinase complex on activated platelets, and thrombin is formed with that complex. This thrombin again activates platelets and coagulation factors (amplification phase), inducing the production of large amounts of FXa and thrombin (propagation phase), resulting in fibrin formation

For considering the anticoagulant mechanism of DOACs, the cell-based coagulation reaction [3] method proposed by Hoffman is relatively useful (Fig. 1). The presence of tissue factor (TF) is revealed in endothelial cells or peripheral monocytes by physicochemical coagulation stimulation, such as endotoxin or immune complex of antiphospholipid antibodies, resulting in the formation of a small amount of thrombin, initial thrombin (initiation phase). Initial thrombin activates nearby platelets and coagulation factors except for fibrinogen. Tenase (X-ase) is then formed with FXIa, FIXa, and FVIIIa on the negative-charged phospholipid membrane of activated platelets, and FXa is converted from FX by X-ase. FXa forms a prothrombinase complex consisting of FVa and FXa on activated platelets, and thrombin is formed by that complex. This thrombin again activates platelets and coagulation factors (amplification phase), inducing the production of large amounts of FXa and thrombin (propagation phase). The large amount of thrombin formed in the propagation phase plays a role to form fibrin, resulting in thrombus formation.

Direct thrombin inhibitors such as dabigatran seem to obstruct X-ase by inhibiting thrombin generated in the initiation phase as well as feedback to the amplification phase of cell-based coagulation reactions. X-ase consists of activated intrinsic coagulation factors; thus, the influence of a direct thrombin inhibitor may be reflected in activated partial thromboplastin time (APTT), which is used as a screening test for intrinsic coagulation factors. On the other hand, inhibitors of FXa are considered to inhibit the activity of factor Xa mainly in the prothrombinase complex of the propagation phase. The effects of Xa inhibitors may be reflected in prothrombin time (PT), used as a screening test for extrinsic and common pathway coagulation factors, as the prothrombinase complex consists of activated common pathway coagulation factors. DOACs are considered to show stronger anticoagulant effects because these agents are able to inhibit X-ase and prothrombinase complex, which are amplified systems in the coagulation reaction. The purpose of the coagulation reaction is to immediately generate excess thrombin with X-ase and prothrombinase. Thus, it seems that anticoagulants primarily act to inhibit these amplification systems.

### Dosage and dose regimen of DOACs for each indication

The dosages and dose regimens of DOACs for VTE prevention and SPAF indication are summarized in Table 2. DOAC dosage and dose regimens have been investigated in phase II as well as phase III studies with dose-adjusted warfarin, which was selected according to phase II results. Safety evaluations have also been performed in an SPAF phase II study, though dose conclusions based on efficacy evaluation are impossible due to the very low incidence of stroke. Therefore, it seems that dosage and dose regimen can be selected based on data from phase II studies for other indications (primarily treatment of DVT and PTE), or by utilizing data from a phase II study of SPAF and then investigating using multiple arms in a phase III study. According to published information, rivaroxaban and apixaban were investigated by the former approach, while dabigatran and edoxaban were investigated using the latter in a three-arm design that compared low and high doses of DOACs with dose-adjusted warfarin.

**Table 2:** Doses of each DOAC for approved indications in Europe, the USA, and Japan

	VTE prevention (V)		SPAF (S)		(S)/(V) daily dose ratio
	Dose	Area	Dose	Area	
Dabigatran	220 mg OD	EU	150 mg BID 110 mg BID	EU, JP	1–1.4
			150 mg BID	US	–
Rivaroxaban	10 mg OD	EU, US	20 mg OD	EU, US	2
			15 mg OD	JP	–
Apixaban	2.5 mg BID	EU	5 mg BID	EU, US, JP	2
Edoxaban	30 mg OD	JP	60 mg OD 30 mg OD	EU, US, JP	2

The dose for edoxaban for SPAF has not yet been confirmed, though results of 30- and 60-mg administrations have been reported in phase III studies

*OD* once daily, *BID* twice daily, *EU* Europe, *US* United States of America, JP: Japan

With dabigatran, a daily dose of 220 mg is used in a twice daily regimen for SPAF, while a once daily regimen has been selected for VTE prevention [4–6]. Indeed, for three of the four available DOACs (edoxaban, rivaroxaban, dabigatran), reduction of trough level using a once daily regimen is recommended for at least VTE prevention. Conversely, with apixaban, a twice daily regimen was reported to be consistently selected for both VTE prevention and SPAF indication, with the rationale apparently based on a comparison conducted in a phase II VTE prevention study [7]. Although the efficacy of 5 mg of apixaban given once daily was nearly comparable to that of 2.5 mg given twice daily, the bleeding incidence trends (major and all bleeding) were not consistent in a comparison between once and twice daily dosages with total daily doses ranging from 5 to 20 mg. Thus, it is possible that the dosage and dose regimen for each DOAC is not based on appropriate or adequate evidence.

### Differences in dose regimens among DOACs and their antithrombotic effects

The anticoagulant activity of warfarin shows no significant diurnal variation, and the agent provides a persistent anticoagulant effect by moderate reductions of several coagulation factors. In terms of inhibition of thrombus formation, persistent anticoagulation throughout the day may be an advantage, though that is associated with an increased risk of bleeding. On the other hand, DOACs show intermittent anticoagulant effects because of their short half-lives. Intermittent anticoagulation may raise a concern about suppression of thrombus formation. A sufficient anticoagulant effect was shown to be obtainable with a once daily regimen of low-molecular-weight heparin agents such as enoxaparin, which have a shorter half-life of only 4 h [8], and the clinical efficacy of intermittent anticoagulation was proven when those were used as prophylaxis for thrombosis.

**Fig. 2:**
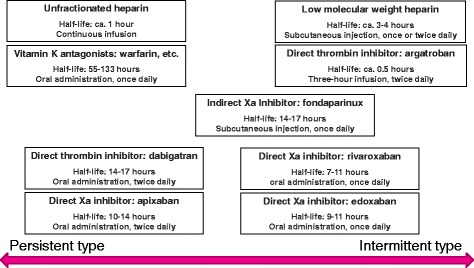
Anticoagulation based on duration of action with consideration of half-life and dose regimen

When a DOAC with a half-life of about 12 h is administered twice daily at 12-h intervals, the next dose is given at around the half-life time point. Theoretically, the plasma concentration at the trough is maintained at a level higher than half of the peak plasma concentration. In practice, a certain level of the plasma concentration area overlaps the next one, which causes a steady increase in the trough level [8]. In other words, if a DOAC is administered with a twice daily regimen, the trough level is increased, and the difference between the peak and trough levels is reduced, which can be interpreted as persistent anticoagulation similar to that seen with warfarin. However, in clinical cases, the anticoagulant effect of apixaban is satisfactorily maintained, without actual accumulation. It is considered that the Ki value of apixaban is lower as compared to other Xa inhibitors, though the detailed reason for this phenomenon is unknown. On the other hand, if a DOAC is administered as a once daily regimen, there is a larger difference between the peak and trough levels. At the trough level, hemostatic activity is nearly normal, as most of the effect of the administered DOAC disappears. This can be interpreted as intermittent anticoagulation similar to that reported for low-molecular-weight heparin, which has quite a short half-life of 4 h and is administered in a once or twice daily regimen. These issues are summarized in Fig. 2.

With intermittent anticoagulation, the risk of thrombus formation during the period of no anticoagulation around the trough should be considered. In normal subjects, physiological coagulation inhibitors, such as tissue factor pathway inhibitor (TFPI) and the thrombomodulin (TM)-protein C system, as well as antithrombin (AT) and fibrinolytic activities likely act to prevent thrombus formation over the endocardium of the left atrial appendage. However, in patients with atrial fibrillation, a sufficient level of physiological anticoagulation does not seem to be obtainable; thus, they tend to have a risk of thrombosis. Interestingly, it was reported that decreased expressions of both mRNA and proteins of TFPI and TM were found in left atrial cells of rapid pacing model rats in comparison with those from healthy rats [9]. On the other hand, other physiological coagulation inhibitors such as AT exists in these patients if TFPI and the TM-protein C system are impeded, and some anticoagulant effects by AT are expected for the prevention of thrombosis and fibrinolytic activity, even in patients with atrial fibrillation. This weak endogenous anticoagulation effect is considered to remain even when the plasma concentration of the administered DOAC is reduced. Therefore, it may enable the use of DOACs in a once daily regimen in which the anticoagulant effect disappears at the trough phase, though the risk of thrombosis increases as compared with that at the peak phase.

With edoxaban, a higher bleeding rate was reported in cases with a twice daily regimen as compared to once at the same daily dose [10]; thus, once daily is recommended for that drug. A once daily regimen is also recommended for rivaroxaban for similar reasons [11, 12]. On the other hand, apixaban showed comparable efficacy and safety with once and twice daily regimens for VTE prevention after total knee arthroplasty [7], as shown in Table 2. With a direct thrombin inhibitor, a once daily regimen is not selected due to inadequate efficacy. While both regimens are available for Xa inhibitors, once daily is recommended for rivaroxaban and edoxaban based on better safety profiles and twice daily for apixaban because of better efficacy.

### Relative potency of DOAC

As noted above, DOAC dosages were determined based on the results of phase I and/or II trials and divided into normal and reduced doses. In addition, the effects were compared with those of warfarin therapy in worldwide large-scale clinical tests. Nevertheless, it is difficult to directly compare the effects among DOACs, though a comparison of each drug with the effect of warfarin therapy is possible. Furthermore, the warfarin therapy groups in those past clinical trials of DOACs were not comprised of the same members and their backgrounds differed, making it difficult to compare the effects of each DOAC. Nevertheless, some indicators are useful to examine DOAC regimens at different dosages; thus, we attempted to compare their potency using drug-related parameters. With an oral medication, the amount of drug that appears in plasma can be approximately calculated using dose, bioavailability, and protein-binding ratio. Furthermore, by adding the inhibition constant to this scheme, the extent of inhibition of the target coagulation factor in blood can be calculated for each DOAC. Using this method, we first calculated the potency of 20 mg of rivaroxaban and then compared potencies based on that of rivaroxaban, with the results shown in Table 3. The profile of each DOAC (molecular weight, bioavailability, protein-binding rate, inhibitory constant) was essentially gathered from the detailed review of Eriksson BI et al. [13]. However, some parameters were not used in that study; thus, the bioavailability of rivaroxaban and molecular weight and bioavailability of edoxaban were obtained from the review of Harbrecht U [1], and the protein-binding rate of edoxaban was obtained from the original report of Ogata K et al. [14]. We initially attempted to convert the potency of an Xa inhibitor in comparison with dabigatran as the standard, though those could not be compared, likely because of their different pharmacological actions, since the volume of thrombin is much larger than that of Xa. Thus, the Ki value of a thrombin inhibitor cannot be compared with that of an Xa inhibitor, since Ki is calculated from the concentrations of free enzymes and free inhibitor, and their complex. As a result, conversion was done using rivaroxaban, the first clinically available Xa inhibitor, as the standard.

**Table 3:** Potency conversion of DOAC

	Rivaroxaban	Dabigatran	Apixaban	Edoxaban
Molecular weight (Da)	436	628	460	548
(a) Molecular weight ratio to rivaroxaban	1	1.44	1.06	1.26
Bioavailability (%)	90*^a^	6.5	66	47.5*^c^
(b) Bioavailability ratio to rivaroxaban	1	13.85	1.36	1.89
Protein binding (%)	93.5*^b^	35	87	49.5*^d^
(c) Protein-binding ratio to rivaroxaban	1	0.37	0.93	0.53
Inhibitory constant; Ki (nM)	0.4	4.5	0.08	0.56
(d) Ki ratio to rivaroxaban	1	11.25	0.20	1.40
(A) Total ratio to rivaroxaban = (a)X(b)X(c)X(d)	1	83.99	0.27	1.77
(B) SPAF customary daily dose (mg)	20	300	10	60
Potency conversion; matching dose for customary dose of rivaroxaban (B)/(A)	20	3.57	37.35	33.99
(C) SPAF reduced daily dose (mg)	15	220	5	30
Potency conversion; matching dose for reduced dose of rivaroxaban (C)/(A)	15	2.62	18.68	17.00

*Average from ^a^80–100, ^b^92–95, ^c^45–50, ^d^40–59

*SPAF* stroke prevention in atrial fibrillation, *Ki* inhibitory constant

We found it interesting that our potency calculation findings showed that the doses of apixaban and edoxaban were comparable even though the dose regimens were different, and much higher than rivaroxaban at its customary dose. With reduced doses, edoxaban was found to be nearly comparable to rivaroxaban; hence, apixaban remained even higher. However, the standard daily dose of DOACs, excluding dabigatran, for SPAF is twice that used for VTE prevention (20 and 10 mg, respectively, for rivaroxaban; 10 and 5 mg, respectively, for apixaban); thus, the daily dosage of edoxaban is most likely to be 60 mg (30 mg when given twice daily). If that is true, it is very interesting that the potency of edoxaban at 60 mg was shown to be similar to that of apixaban at 10 mg despite the different dose regimens and doses selected from various clinical studies.

Although the dose of apixaban is 5 mg, lowest among available DOACs, its anticoagulant efficacy seems to be greater than that of rivaroxaban and edoxaban when based on this comparison. Moreover, our findings suggest that apixaban therapy can maintain a persistent level of anticoagulation similar to that shown by warfarin by administering as a twice daily regimen, which provides more than 90 % of the potency of rivaroxaban (equivalent to 18.68 mg of rivaroxaban twice daily), while it also maintains an intensity close to the peak obtained with a once daily regimen of rivaroxaban. This would suggest that the level of anticoagulation provided by apixaban is quite different from that by rivaroxaban. In fact, even a reduced dose of apixaban (2.5 mg twice daily) nearly matches the customary dose of 20 mg of rivaroxaban given daily (18.68 mg). With edoxaban, when 60 mg is given, the potency is higher than that of rivaroxaban and lower than that of apixaban, while the potency of a 30-mg dose is intermediate between the normal and reduced dose of rivaroxaban. A comparison of the potency of reduced doses of three different Xa inhibitors showed a lower level of difference as compared with normal doses. However, the dose reduction criteria in each study were different, and the percentage of subjects in the dose-reduced group receiving apixaban was different as compared to those in the rivaroxaban and edoxaban groups (4.7, 21.0, and 25.3 %, respectively) [15–17], Therefore, the interpretation of findings for comparison of potency of a reduced dose is not possible at this time.

As described above, the present comparison of potencies may contribute to a better understanding of the purpose of anticoagulant therapy, which can be adjusted by selecting either a once or twice daily regimen. However, since the only data available were obtained under varying circumstances during drug development phases, clinical outcomes cannot be directly compared at this time and should not be considered accurate. In addition, it is not yet clear which DOAC or regimen is better for a specific condition. With additional accumulation of clinical findings, comparisons in regard to persistent and intermittent anticoagulation will be possible.

## Conclusions

It is thought that the numbers of patients receiving DOACs will increase considerably in the near future. Therefore, additional information about these drugs and their use for anticoagulant therapy is considered to be necessary for physicians working in the ICU. As it was difficult to directly compare the effects among DOACs, we attempted to compare their potency using drug-related parameters. With our results, the present comparison of potencies may contribute to a better understanding of the purpose of anticoagulant therapy.
